# The role of MYB34, MYB51 and MYB122 in the regulation of camalexin biosynthesis in *Arabidopsis thaliana*

**DOI:** 10.3389/fpls.2015.00654

**Published:** 2015-08-25

**Authors:** Henning Frerigmann, Erich Glawischnig, Tamara Gigolashvili

**Affiliations:** ^1^Botanical Institute and Cluster of Excellence on Plant Sciences, University of Cologne, CologneGermany; ^2^Lehrstuhl für Genetik, Technische Universität München, FreisingGermany

**Keywords:** camalexin biosynthesis, transcriptional regulation, MYB51, MYB122, MYB34

## Abstract

The phytoalexin camalexin and indolic glucosinolates share not only a common evolutionary origin and a tightly interconnected biosynthetic pathway, but regulatory proteins controlling the shared enzymatic steps are also modulated by the same R2R3-MYB transcription factors. The indolic phytoalexin camalexin is a crucial defense metabolite in the model plant *Arabidopsis*. Indolic phytoalexins and glucosinolates appear to have a common evolutionary origin and are interconnected on the biosynthetic level: a key intermediate in the biosynthesis of camalexin, indole-3-acetaldoxime (IAOx), is also required for the biosynthesis of indolic glucosinolates and is under tight control by the transcription factors MYB34, MYB51, and MYB122. The abundance of camalexin was strongly reduced in *myb34/51* and *myb51/122* double and in triple *myb* mutant, suggesting that these transcription factors are important in camalexin biosynthesis. Furthermore, expression of *MYB51* and *MYB122* was significantly increased by biotic and abiotic camalexin-inducing agents. Feeding of the triple *myb34/51/122* mutant with IAOx or indole-3-acetonitrile largely restored camalexin biosynthesis. Conversely, tryptophan could not complement the low camalexin phenotype of this mutant, which supports a role for the three MYB factors in camalexin biosynthesis upstream of IAOx. Consistently expression of the camalexin biosynthesis genes *CYP71B15/PAD3* and *CYP71A13* was not negatively affected in the triple *myb* mutant and the MYBs could not activate *pCYP71B15::uidA* expression in *trans*-activation assays with cultured *Arabidopsis* cells. In conclusion, this study reveals the importance of MYB factors regulating the generation of IAOx as precursor of camalexin.

## Introduction

**FIGURE 1:**
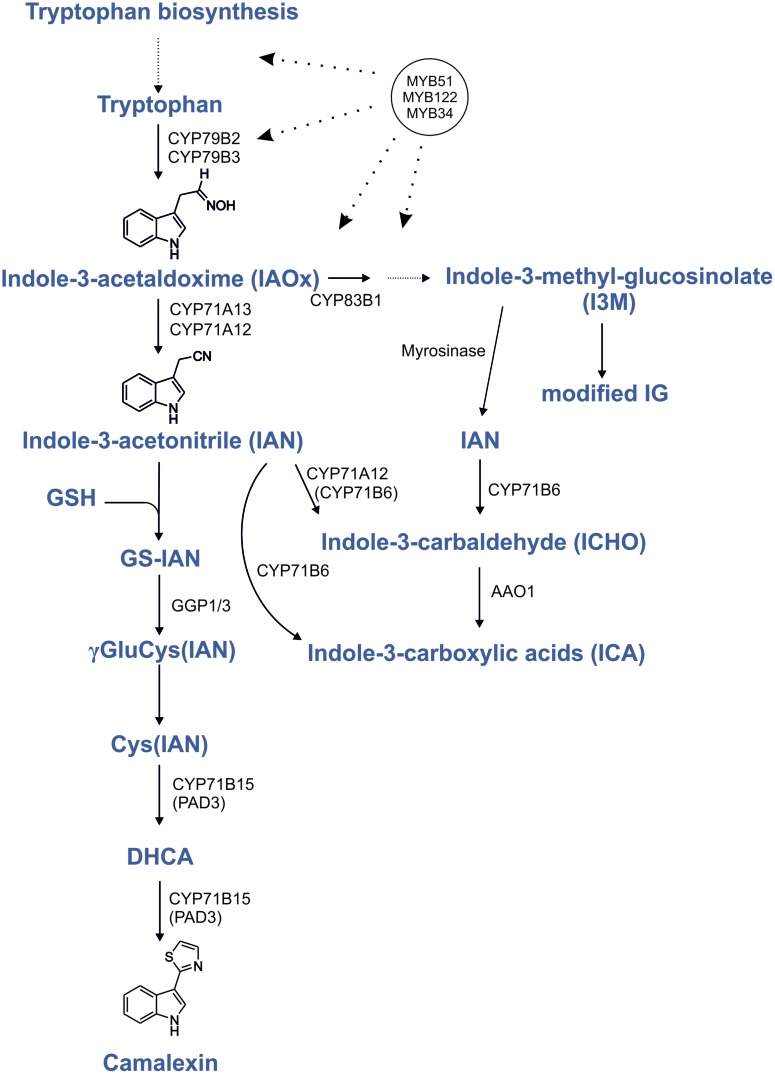
**Regulation by MYB transcription factors in the camalexin biosynthesis pathway.** Proven positive transcriptional regulation is shown by dotted lines with arrows. Modified from Böttcher et al. (2009), Geu-Flores et al. (2011), and Müller et al. (2015); indolic glucosinolates (IG); glutathione (GSH); dihydrocamalexic acid (DHCA).

Phytoalexins are important defense compounds produced by plants in response to infection by a large variety of microorganisms. However, the elucidation of camalexin biosynthesis benefited from the fact that abiotic elicitors like silver nitrate (AgNO_3_; Glawischnig et al., 2004) and UV (Müller et al., 2015) strongly induce the camalexin production. Camalexin (3-thiazol-2′-yl-indole) is an indole alkaloid phytoalexin that is specific to a group of cruciferous species including the model plant *Arabidopsis thaliana*, but is absent in more distantly related *Brassicaceae* species (Glawischnig, 2007; Rauhut and Glawischnig, 2009; Bednarek et al., 2011). The induction of camalexin biosynthesis genes is strictly localized to sites of pathogen application, as demonstrated by quantitative RT-PCR and reporter-gene analysis and there is no evidence existing for long-distance camalexin transport (Schuhegger et al., 2007). During camalexin biosynthesis, tryptophan (Trp) is converted to indole-3-acetaldoxime (IAOx; **Figure 1**). This step is shared with the biosynthesis of other Trp-derived metabolites and is catalyzed by two homologous cytochrome P450 enzymes, CYP79B2, and CYP79B3 (Hull et al., 2000; Mikkelsen et al., 2000; Zhao et al., 2002). The resulting IAOx is a precursor of camalexin, indolic glucosinolates (IGs) and indole-carboxylic acids (ICAs; Böttcher et al., 2014). In camalexin biosynthesis, IAOx is dehydrated to indole-3-acetonitrile (IAN) by CYP71A12 and CYP71A13 (**Figure 1**; Müller et al., 2015). In accordance with their specific function in phytoalexin biosynthesis, both corresponding genes are expressed at very low levels in the absence of stress and are induced by pathogen infection, application of pathogen-associated molecular patterns (PAMPs), or by AgNO_3_ (Nafisi et al., 2007; Millet et al., 2010). IAN is also generated during the degradation of glucobrassicin (I3M; Burow et al., 2008) and it can be converted to indole-3-carbaldehyde (ICHO) and ICA by CYP71B6 (Böttcher et al., 2014) (**Figure 1**). Under specific conditions IAN serves also as a precursor for IAA (Kutz et al., 2002; Park et al., 2003). However, the IAN pools seem to be strictly seperated, thus IAN from I3M breakdown cannot serve as a precursor of camalexin, but only for ICAs, as it was shown with a TALEN generated *cyp71A12 cyp71a13* double knockout (Müller et al., 2015). Notably, the *cyp79b2/b3* double knockout mutant cannot synthesize camalexin (Zhao et al., 2002;Glawischnig et al., 2004), but this ability was recovered in a chemical complementation strategy by feeding the mutant with camalexin precursors such as IAN and dihydrocamalexic acid (DHCA; Schuhegger et al., 2006; Nafisi et al., 2007; Böttcher et al., 2009). In camalexin biosynthesis IAN is conjugated with glutathione (Nafisi et al., 2007; Parisy et al., 2007; Böttcher et al., 2009; Su et al., 2011). From this glutathione conjugate (GS-IAN) a cysteine conjugate Cys(IAN) is formed, involving γ-Glutamyl Peptidases 1 and 3 (GGP1/3; Geu-Flores et al., 2011), which is the substrate for CYP71B15/PAD3 (Zhou et al., 1999; Schuhegger et al., 2006; Böttcher et al., 2009).

Although the pathway leading to camalexin has been largely elucidated, its regulation remains less well understood. Perception of fungal pathogens such as *Botrytis cinerea* (Kliebenstein et al., 2005) and *Alternaria alternata* (Schuhegger et al., 2007) significantly activates camalexin production via mitogen-activated protein kinase (MAPK) cascade, which in turn phosphorylates MPK3 and MPK6 (Ren et al., 2008). Camalexin synthesis is almost completely blocked in the *mpk3/6* double mutant after infection by *B. cinerea* (Ren et al., 2008). Mao et al. (2011) have demonstrated that the *Arabidopsis* transcription factor WRKY33 is a molecular target of the MPK3/6 cascade. *wrky33* mutant can synthesize only very low amounts of camalexin, even in the MPK3/6 gain-of-function mutant. Furthermore, MPK4 physically interacts with MPK4 SUBSTRATE 1 (MKS1) and WRKY33 and represses WRKY33 function. Activated MPK4 phosphorylates MKS1, which in turn, releases WRKY33, which can then bind to the promoter of *CYP71B15* (Qiu et al., 2008). Surprisingly, the respective *wrky33* knock-out mutant contains low camalexin levels only at early stages of infection, but at later stages, contains even more camalexin than wild-type (WT) plants (Birkenbihl et al., 2012). Together, these results indicate that WRKY33 is one important regulator of camalexin, but that other regulators exist.

The transcription of *NAC042* is strongly induced by AgNO_3_, a known inducer of camalexin biosynthesis, and the *nac042* null mutant accumulates about 50% of WT camalexin levels after treatment with AgNO_3_ or *B. cinerea* (Saga et al., 2012). Furthermore, the induction of camalexin biosynthesis by acifluorfen, which generates reactive oxygen species (ROS), results in about 15% of the WT camalexin level in *nac042*, which highlights the key role of *NAC042* in the ROS-dependent induction of camalexin biosynthesis (Saga et al., 2012).

To synthesize camalexin, it is essential that the specific genes (*CYP71A12*, *CYP71A13*, and *CYP71B15*) are upregulated together with the upstream Trp biosynthetic genes and *CYP79B2*. The known regulator of camalexin, WRKY33, binds to the promoters of *CYP71B15* and *CYP71A13* (Birkenbihl et al., 2012), whereas the regulators of IG biosynthesis, MYB34, MYB51, and MYB122 control genes of the shikimate pathway to Trp, i.e., anthranilate synthase α and β subunits, Trp synthases and *CYP79B2* (Gigolashvili et al., 2007; Malitsky et al., 2008; Frerigmann and Gigolashvili, 2014). These MYB transcription factors thus positively regulate all the necessary steps for the production of the camalexin precursor IAOx. In addition to this intermediate, IG and camalexin biosynthesis share a glutathione conjugation step and the involvement of GGP1 (Geu-Flores et al., 2011), reflecting that camalexin biosynthesis likely has evolved from IG biosynthesis (Rauhut and Glawischnig, 2009; Bednarek et al., 2011). Therefore, MYB34, MYB51, and MYB122 possibly not only regulate the IG biosynthesis pathway, but also activate genes in the closely related camalexin biosynthesis pathway. Here we addressed the potential involvement of three MYB transcription factors in camalexin biosynthesis and show that especially MYB51 and MYB122 are involved in camalexin biosynthesis, because its synthesis is strongly reduced in corresponding double and triple mutants. Metabolite complementation of the triple *myb34/51/122* mutant reveals the importance of these MYBs in the regulation of camalexin biosynthesis upstream of IAOx. Thus, camalexin and IGs not only possess a tightly interconnected biosynthetic pathway, but are at least partially regulated by the same R2R3-MYB transcription factors.

## Results

### Camalexin Biosynthesis Genes are Co-expressed with *MYB51* and *MYB122*

Camalexin biosynthesis is induced locally by exposure to biotic or abiotic stresses and the genes involved in its biosynthesis are highly co-ordinately expressed. To address the role of MYB34, MYB51, and MYB122 in camalexin biosynthesis, we exploited existing co-expression databases like ATTED^1^ (Obayashi et al., 2009). The survey revealed that *MYB51* and *MYB122* are not only co-regulated with genes for Trp and IAOx biosynthesis, but also with *CYP71B15/PAD3*, *CYP71A12*, and *CYP71A13* (Supplementary Tables S1 and S2). This implicates both MYB factors as good candidate regulators of camalexin biosynthesis in *Arabidopsis*.

### *MYB51* and *MYB122* are Induced by Silver Nitrate and by Pathogen-Associated Molecular Pattern (PAMP) from *Pythium aphanidermatum* (*PaNie*)

**FIGURE 2:**
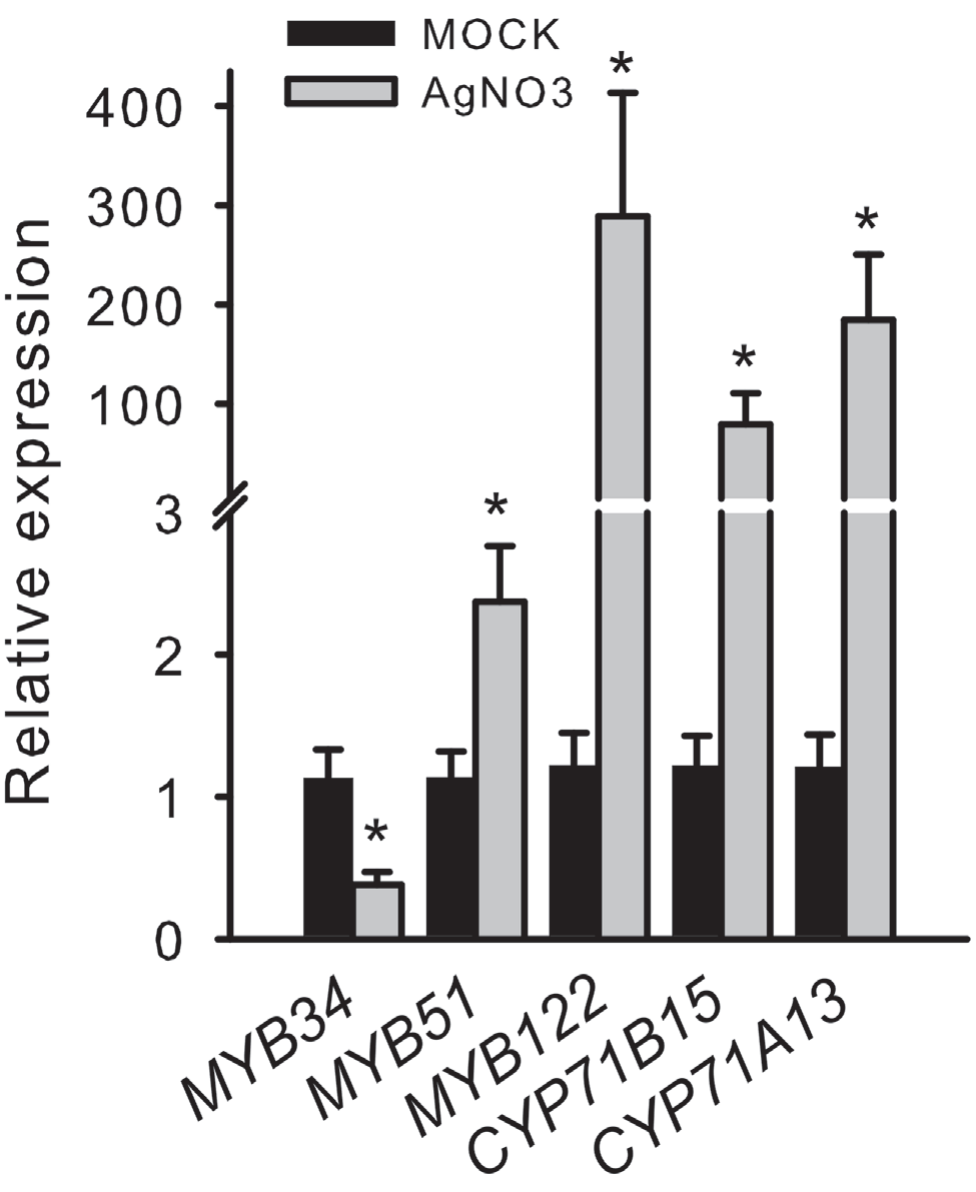
**Silver nitrate induces the transcription of *MYB51* and *MYB122* as well as that of *CYP71B15* and *CYP71A13.*** The expression of camalexin biosynthesis genes (*CYP71B15* and *CYP71A13*) and of *MYB34*, *MYB51*, and *MYB122* upon silver nitrate (AgNO_3_) treatment is shown. The relative expression in Col-0 was measured in leaves of 6-week-old plants 18 h after treatment (MOCK = 1). Data are means ± SE from four independent experiments each with two to three biological replicates (*n* = 11). Values marked with asterisks are significantly different from those of control plants (Student’s *t*-test; *p* < 0.05).

To further validate the importance of R2R3-MYBs in camalexin regulation, we analyzed the induction of *MYB34*, *MYB51*, and *MYB122* in response to elicitors of camalexin production. In a pilot experiment, we treated *Arabidopsis* Col-0 WT plants with AgNO_3_, a commonly used abiotic elicitor of camalexin induction, which strongly induced *CYP71B15*, *CYP71A13*, *MYB122*, and *MYB51* (**Figure 2**). However, the expression of *MYB34* was reduced, indicating that it plays a less important role in phytoalexin regulation.

**FIGURE 3:**
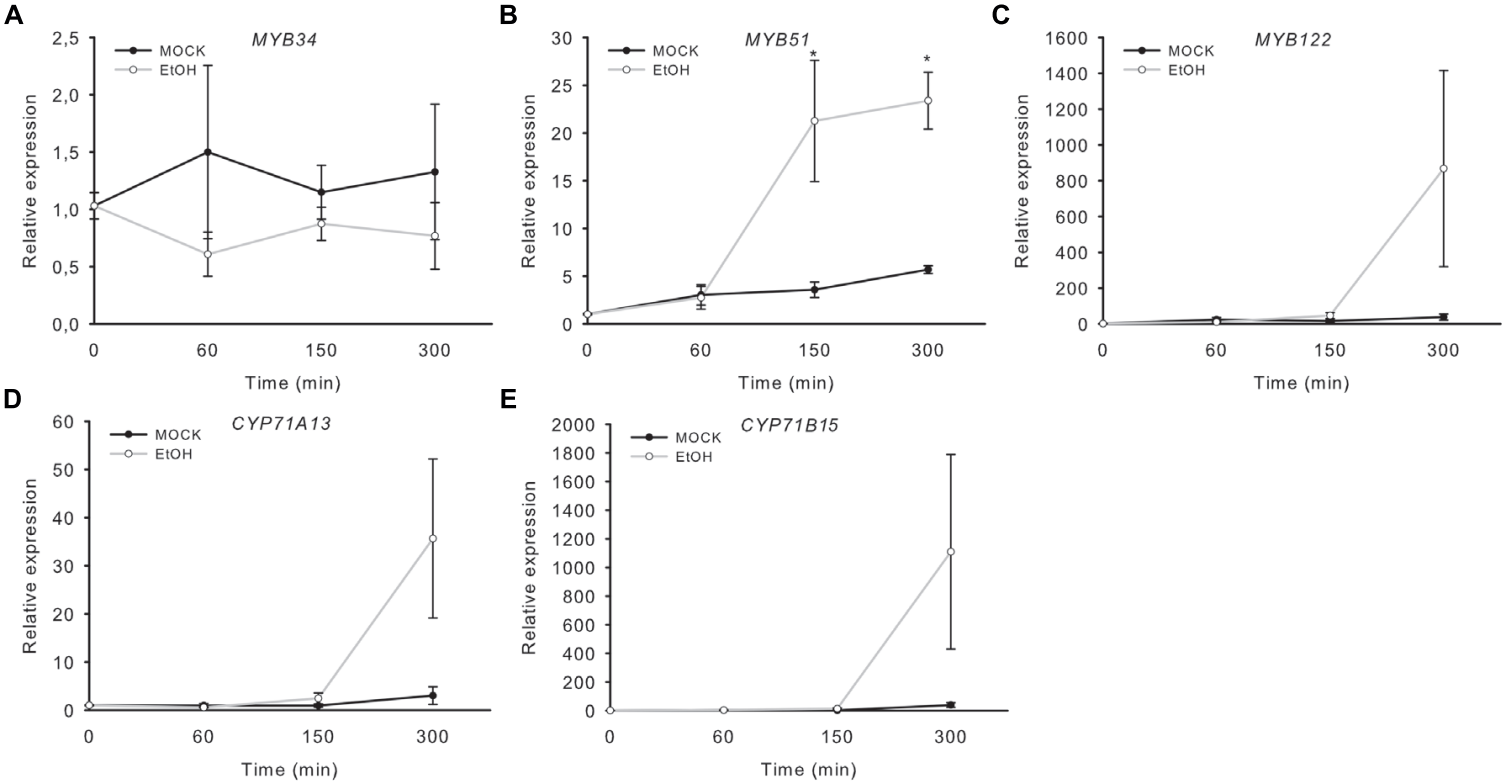
**Induction of *MYB51* and *MYB122* in rosette leaves of *Alc::PaNie_Dc_* plants.** Expression of *MYB34* **(A)**, *MYB51* **(B)**, *MYB122* **(C)**, *CYP71A13* **(D)**, and the camalexin biosynthesis gene *CYP71B15* **(E)** following AgNO_3_ treatment. Relative expression in *pAlc::PaNie_Dc_* was measured in leaves of 6-week-old plants induced with ethanol (for 60 min, 150 min or 300 min; time point 0 = 1 min). Data are means ± SE from two independent experiments each with three biological replicates (*n* = 6). Values marked with asterisks are significantly different from those of control plants (Student’s *t*-test; *p* < 0.05).

In addition, we analyzed transgenic plants that expressed a gene encoding a Nep1-like protein from *Pythium aphanidermatum* (*PaNie*), which acts as a PAMP, under the control of an ethanol-inducible promoter (Rauhut et al., 2009). The production of this Nep1-like protein triggers the strong accumulation of camalexin 8 h following ethanol inductions (Rauhut et al., 2009). The transcription of *MYB51* significantly increased 150 min after treatment, and that of *CYP71B15*, *CYP71A13*, and *MYB122* after 300 min (5 h; **Figure 3**). Conversely, *MYB34* was not induced by *PaNie* expression, which confirms its minor role in camalexin regulation. A similar induction pattern of the MYBs and camalexin genes was observed upon colonization with the fungus *Piriformospora indica* (Lahrmann et al., 2015). In addition, *MYB51* transcription was also increased 40 and 88 h after infection with the necrotrophic pathogen *B. cinerea*, as revealed by the *pMYB51::GUS* reporter (Supplementary Figure S1).

Taken together, the expression patterns of *MYB51* and *MYB122* implicate a role in camalexin biosynthesis.

### The Induction of *MYB51* and *MYB122* upon Wounding Coincides with that of the Camalexin Biosynthesis Gene *CYP71B15*

Wounding of the plant surface creates a potential entry point for invading pathogens, and plant response to injury by localized defense responses includes the induction of defense-related genes and the accumulation of anti-microbial proteins such as proteinase inhibitors, chitinase, or glucosinolates (Ryan, 1990; Chang et al., 1995; Reymond et al., 2000; Chassot et al., 2008). Especially strong wounding releases oligogalacturonides from the plant cell wall which can induce a local defense response similar to bacterial PAMPs (Denoux et al., 2008). Thus wounding of *Arabidopsis* leaves has been previously shown to lead to immunity to *B. cinerea*, because hyphal growth on wounded plants was significantly inhibited in comparison to that on unwounded controls (Chassot et al., 2008).

**FIGURE 4:**
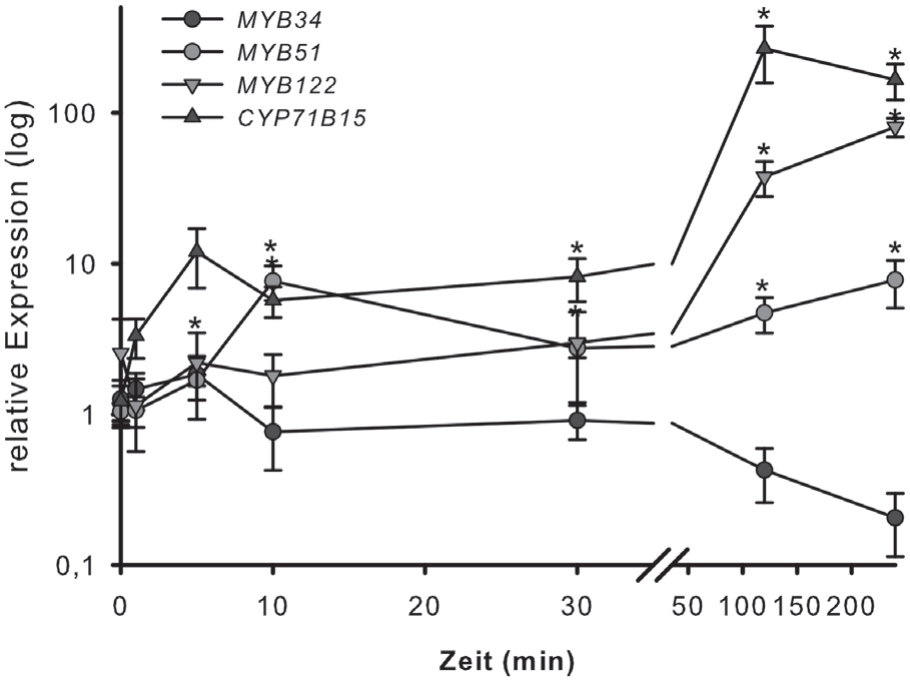
**Wounding response of *MYB34*, *MYB51*, and *MYB122* in leaves.** Detached leaves of 6-week-old Col-0 plants grown under short day conditions were strongly wounded. Leaves were harvested after 1, 5, 10, 30, 120, and 300 min and processed for transcript analysis by qPCR. Relative transcript levels for *MYB34, MYB51*, *MYB122*, and *CYP71B15* are shown for wounded vs. unwounded leaves (time-point 0 = 1 min). Data are means ± SE from three independent cultivations each with two biological replicates (*n* = 6). Values marked with asterisks are significantly different from the 0 time point (Student’s *t*-test; *p* < 0.05).

To address the involvement of the MYB34, MYB51, and MYB122 transcription factors in wounding response, the transcription of their respective genes was analyzed 1, 5, 10, 30, 120, and 300 min after strong wounding. Wounding of WT *Arabidopsis* leaves increased the transcription of *MYB51* and *MYB122*, but not of *MYB34* after 120 min of injury (**Figure 4**), which represented the time-point of increased expression of the camalexin biosynthesis gene *CYP71B15* (**Figure 3**). This second induction peak of *MYB51* and *MYB122* transcription appears to be related to induction of camalexin biosynthesis. During the second phase of the wounding response, the transcript levels of *MYB34* decreased, whereas expression of *MYB51*, *MYB122*, and *CYP71B15* continued to increase and remained high even at 300 min (5 h) of treatment (**Figure 4**). The first peak in *MYB* transcription recorded after 5–10 min of injury observed in this study (**Figure 3**) and previously (Gigolashvili et al., 2007), was associated with an increase in IG biosynthesis.

In order to confirm that the applied strong wounding does not resemble solely jasmonate signaling, as it is known for standard wounding application, hormone marker genes for jasmonate (*VSP2*), salicylic acid (*PR1*), and ethylene/jasmonate (*PDF1.2*) were checked (Supplementary Figure S2). As expected no induction, but even a repression of *VSP2* was observed, while *PR1* and *PDF1.2* transcript levels increased similar to oligogalacturonide treatment (Denoux et al., 2008).

Together, these data suggest a role for MYB51 and MYB122 in priming camalexin biosynthesis at later stages of wounding response, to protect against biotic and abiotic stressors.

### *myb* Mutants are Impaired in UV-Dependent Camalexin Induction

**FIGURE 5:**
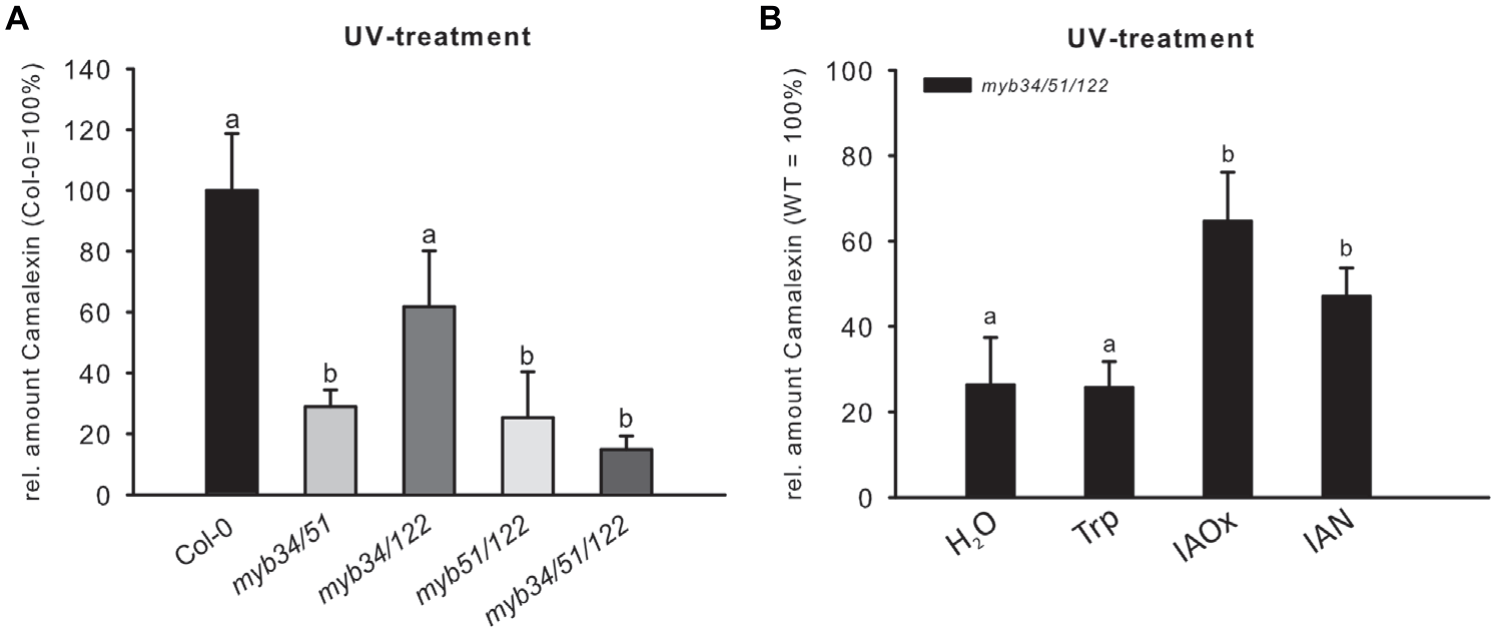
**The UV-dependent induction of camalexin is impaired in multiple *myb* knock-out mutants. (A)** The relative amount of camalexin after 18 h UV treatment in Col-0 and double and triple *myb* mutants (Col-0 = 100%). Data are means ± SE from two independent cultivations each with six biological replicates (*n* = 12). **(B)** The relative amount of camalexin 18 h after UV treatment in *myb34/51/122* fed with H_2_O, or 0.25 mM Trp, IAOx or IAN (Col-0 = 100%). Data are means ± SE from three independent cultivations each with six biological replicates (*n* = 18). Different letters indicate significant differences at *p* < 0.05 (Kruskal Wallis Test, followed by a Mann Whitney *U* Test with Bonferroni-corrected *p-*values; *p* < 0.05).

The abiotic elicitor UV can be easily applied to uniformly trigger camalexin induction in Col-0 (Müller et al., 2015 and Supplementary Figure S3B). We tested double and triple loss-of-function mutants of *MYB51*, *MYB122*, and *MYB34* for their ability to synthesize camalexin after UV treatment. The camalexin content of leaves of double *myb51/122*, *myb34/51*, and triple *myb34/51/122* mutants was strongly reduced after 18 h UV treatment (**Figure 5**), suggesting an important function for all three MYBs and especially MYB51 in camalexin accumulation. The *myb34/122* double mutant showed only a minor and statistically non-significant reduction in camalexin accumulation. Camalexin levels were significantly lower in the triple *myb34/51/122* mutant than in WT plants, but not in comparison to that of the *myb34/51* and *myb51/122* mutants. However, 24 h after UV treatment, only the *myb51/122* double mutant and *myb34/51/122* triple mutant contained significantly less camalexin than the WT (Supplementary Figure S3). These camalexin levels in *myb* mutant backgrounds confirm the importance of *MYB51* and *MYB122* in camalexin accumulation. The role of *MYB34* appears to be minor.

### The Camalexin Biosynthesis Genes *CYP71B15* and *CYP71A13* are not Downregulated in the Triple *myb34/51/122* Mutant

The *myb34/51/122* triple mutant is limited in the synthesis of IAOx, a precursor of IGs and camalexin and consequently, IGs (Frerigmann and Gigolashvili, 2014) and camalexin are reduced (**Figure 5A**). To investigate the role of MYBs on the expression of camalexin biosynthesis genes, the steady-state mRNA levels of *CYP71B15* and *CYP71A13* were analyzed in the triple *myb34/51/122* mutant. If MYB51, MYB122, and MYB34 directly regulate camalexin biosynthesis genes, the expression of these genes should be significantly decreased in *myb34/51/122*, similar to that of IG biosynthesis genes.

**FIGURE 6:**
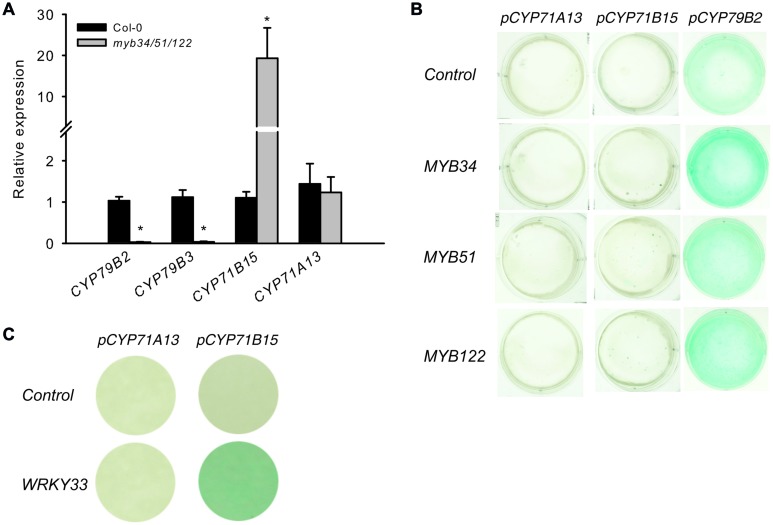
**The presence of solely *MYB34*, *MYB51*, or *MYB122* is not enough to activate the *pCYP71B15:uidA* and *pCYP71A13:uidA in trans.* (A)** The expression of specific genes for camalexin biosynthesis (*CYP71B15* and *CYP71A13*) and genes underlying the conversion of tryptophan to IAOx (*CYP79B2* and *CYP79B3*) was analyzed in the *myb34/51/122* mutant. Relative expression was measured in leaves of 6-week-old plants (Col-0 = 1). Data are means ± SE from three independent cultivations with three biological replicates (*n* = 9). Values marked with asterisks are significantly different from those of control plants (Student’s *t*-test; *p < 0.05). **(B,C)** *Trans*-activation with *MYB34*, *MYB51*, and *MYB122* and target promoters of the camalexin biosynthesis pathway genes *CYP79B2, CYP71A13*, and *CYP71B15*. **(B)** The promoter–reporter constructs of *pCYP71A13:uidA*, *pCYP71B15:uidA* or *pCYP79B2:uidA* were co-expressed in the same cells with effector constructs *p35S:MYB34*, *p35S:MYB51*, or *p35S:MYB122*. The cultured *A. thaliana* cells were inoculated with the supervirulent *Agrobacterium tumefaciens* strain *LBA4404.pBBR1MCS.virGN54D*, containing either only the reporter construct or the reporter and effector construct in a 1:1 ratio. The GUS staining indicates *trans*-activation of the promoter by the effector. **(C)** The *trans-*activation potential of the *p35S:WRKY33* effector toward the promoters of *CYP71A13* and *CYP71B15*.

Genes involved in IAOx biosynthesis were strongly down regulated in the *myb34/51/122* mutant (**Figure 6**), whereas the expression of *CYP71B15* and *CYP71A13* either remained unchanged or increased. This increase in specific camalexin gene expression is not accompanied by higher levels of camalexin in the mutants (Supplementary Figure S3). The activity of *pCYP79B2:uidA* increased, whereas that of *pCYP71B15:uidA* was not affected by all three MYB factors, as demonstrated by co-expression via *trans*-activation assays with cultured *Arabidopsis* cells (Berger et al., 2007) (**Figure 6B**). Conversely, *WRKY33*, the transcription regulator of *CYP71B15*, induced *pCYP71B15::uidA* when co-expressed with *p35S:WRKY33* in cultured cells. Thus, MYBs do not directly regulate these important camalexin biosynthesis genes downstream of IAOx.

We also attempted to metabolically complement the low-camalexin phenotype of *myb34/51/122* mutant leaves upon UV-treatment, by feeding them with IAOx, IAN or Trp. Treatment with IAOx or IAN partially restored camalexin levels in the *myb34/51/122* mutant upon UV-treatment, whereas Trp feeding did not (**Figure 5B**). Because Trp could not complement the low-camalexin phenotype of the triple *myb* mutant, we conclude that the three MYB factors studied essentially regulate the synthesis of IAOx from Trp, but are not directly involved in the activation of genes downstream of IAOx.

## Discussion

The camalexin biosynthetic pathway has been largely elucidated, but little is known about the regulatory components of this pathway. *WRKY33* binds to the promoters of *CYP71B15* and *CYP71A13* to activate camalexin biosynthesis, but also other regulators have to be involved, because its loss of function leads to low camalexin levels only during early stages of pathogen infection (Birkenbihl et al., 2012). In this study, we addressed the role of the known IG regulators MYB34, MYB51, and MYB122 in the biosynthesis of camalexin in *Arabidopsis*. Because the camalexin and IG biosynthetic pathways have a common evolutionary origin and are tightly interconnected, these two classes of compounds might be regulated by the same set of transcription factors.

### *MYB51* and *MYB122* are Induced by Biotic and Abiotic Triggers of Camalexin Biosynthesis

Camalexin biosynthesis is induced in plants following exposure to abiotic stresses such as heavy metal treatment or UV-C radiation or exposure to pathogens. We addressed the role of MYB34, MYB51 and MYB122 in camalexin biosynthesis by analysing their mRNA levels in plants exposed to several camalexin-inducing agents. Treatment of *Arabidopsis* WT plants with the abiotic elicitor AgNO_3_ caused a significant increase in steady-state mRNA levels of *MYB122* and *MYB51*, but not of *MYB34.* Similarly, *MYB122* and *MYB51* were induced in transgenic plants that expressed a NEP1-like protein from *PaNie* under the control of an ethanol-inducible promoter (Rauhut et al., 2009), endorsing the possible role of these two transcription factors in camalexin biosynthesis. In addition, the *MYB51* promoter was also induced after treatment with *B. cinerea* (Supplementary Figure S1). Finally, wounding of leaves, which is known to provide a protection against *B. cinerea* by priming camalexin production in *Arabidopsis* (Chassot et al., 2008), increased the expression of *MYB51* and *MYB122*. Two induction peaks of *MYB* expression in response to wounding within the analyzed time scale occurred: the first peak in wounding response of *MYB51* transcript level was observed after 5–10 min of injury (Gigolashvili et al., 2007) and was associated with a switch in the IG biosynthesis machinery, and the second phase of induction concerned the transcription of *MYB51* and *MYB122*, but not *MYB34*, after 120 min of injury, which coincided with an strongly increased expression of the camalexin biosynthesis gene *CYP71B15* (**Figure 3**). This second phase might therefore be related to camalexin biosynthesis. According to directed studies (Schuhegger et al., 2006; Chassot et al., 2008) and to the analysis of microarray data (see, e.g., efp browser analysis of ATH1 Affymetrix data^2^; Winter et al., 2007) *CYP71B15* did not show strong responsiveness to wounding. In this light it was surprising that here *CYP71B15* expression was induced more than 100-fold. We here applied rather harsh and extensive wounding to the tissue. Possibly severe wounding induces camalexin biosynthesis by eliciting oligogalacturonides, which originate from the plant cell wall (Denoux et al., 2008), while restricted wounding has a minor effect.

Taken together, the induced expression of *MYB51* and/or *MYB122* after exposure to biotic and abiotic triggers of camalexin biosynthesis [AgNO_3_, wounding, PAMP (*PaNie*) and the necrotrophic pathogen *B. cinerea*] suggests that the transcription factors encoded by these genes play a role in camalexin biosynthesis. Because the expression of *MYB34* was not affected by the same treatments, we conclude that it is not involved in camalexin biosynthesis.

### The Role of MYBs in the Regulation of IAOx – A Branch-Point in IG, Camalexin, ICA, and IAA Synthesis

The initial step of camalexin, IG, and ICA biosynthesis is the conversion of Trp to IAOx mediated by CYP79B2 and CYP79B3. The interplay between IAOx-derived metabolites was also demonstrated by the analysis of mutants deficient in IG biosynthesis. The loss of function of IG biosynthetic genes downstream of IAOx (*cyp83b1/sur2*, *C-S lyase/sur1*, and *ugt74b1* null mutants) results in a strong auxin-overproducing phenotype (Barlier et al., 2000; Grubb et al., 2004; Mikkelsen et al., 2004). This is possibly due to IAOx accumulation in cells because of “biosynthetic blockage” in the IG pathway, and consequently, unspecific conversion of excess IAOx to the auxin IAA. Other metabolites such as ICA and camalexin, which can be also induced in these mutants, were not analyzed in the above-mentioned studies.

In the WT, biosynthesis of IAOx is under tight transcriptional control by MYB34, MYB51 and MYB122 transcription factors (Celenza et al., 2005; Gigolashvili et al., 2007; Frerigmann and Gigolashvili, 2014). Consequently the MYBs, especially the MYB51 and MYB122 are considered as candidates in the regulation of other Trp-derived metabolites than IGs, e.g., camalexin. We propose the following scenario for the role of MYB34, MYB51, and MYB122 in camalexin biosynthesis: they regulate genes involved in camalexin biosynthesis similar to how they regulate IG production. However, they have to act in concert with other regulators, since they are also highly expressed and regulate IG production in non-triggered tissue and would therefore lead to camalexin accumulation in healthy plants. We therefore suggest that specific signaling components exist upstream to these MYB factors, including alternative transcription factors, which activate different sets of genes for camalexin and IG biosynthesis. These different signaling components are responsive to AgNO_3_, PAMPs, and UV in the case of camalexin biosynthesis, and to herbivores regarding IG production. Thus, to enable camalexin biosynthesis, the MYBs and additional transcription factors are activated: MYB factors regulate IAOx biosynthesis, and alternative (unknown) regulators, together with WRKY33, control camalexin genes downstream of IAOX (e.g., *CYP71B15* and *CYP71A13*).

### The Regulation of Camalexin Biosynthesis by MYB51, MYB122, and MYB34

The analysis of camalexin accumulation in higher-order loss-of-function mutants of *MYB51, MYB122*, and *MYB34* treated with UV revealed a strong reduction in the camalexin content of leaves of double *myb51/122, myb34/51*, and triple *myb34/51/122* mutants (**Figure 5**), emphasizing the importance of MYB51 in camalexin accumulation in *Arabidopsis*. The role of MYB34 for camalexin induction was negligible, whereas MYB122 contributes camalexin biosynthesis, as demonstrated by the response of higher-order *myb* mutants after 24 h treatment with UV (Supplementary Figure S3). We propose the following explanation for the observed role of *MYB122*: (i) *MYB122* is the lowest-expressed gene among the three MYBs (Frerigmann and Gigolashvili, 2014), therefore, the observed metabolic effects reflect its transcript abundance; (ii) transcription of *MYB122* is positively regulated by *MYB51*, which is essential for camalexin biosynthesis. This positive correlation between *MYB51* and *MYB122* expression due to reciprocal regulation was previously demonstrated by the analysis of *myb* knock-out and overexpression plants (Frerigmann and Gigolashvili, 2014). The reciprocal activation of mRNAs of these two MYBs might play an important role in the regulation of camalexin biosynthesis.

To elucidate further the role of the MYBs, we performed a metabolic complementation experiment by feeding the UV-treated leaves of the camalexin-deficient mutant *myb34/51/122* with the precursors Trp, IAOx, or IAN (**Figure 5B**). This experiment demonstrated that the three MYBs are essential to regulate the synthesis of IAOx from Trp during camalexin biosynthesis. However, they are not directly involved in the activation of genes downstream of IAOx, because both IAOx and IAN could complement the low camalexin phenotype of the *myb34/51/122* mutant. These experiments suggest the possibility that MYB51 and MYB122 are indirectly involved in the activation of *CYP71B15* or *CYP71A13* by forming dynamic regulatory complexes with other transcription factors. However, even if the MYB factors interact with other transcription factors that regulate camalexin biosynthesis, they are not thought to activate *CYP71B15* or *CYP71A13*. In support of this, qRT-PCR analysis of the triple *myb34/51/122* mutant and the promoter–effector assays in cultured cells suggested that *CYP71B15* and *CYP71A13* are regulated independently from the *MYB* genes (**Figure 6**).

Taken together, the data substantiate the importance of three MYB factors in the regulation of camalexin biosynthesis by providing the precursor metabolite IAOx (**Figure 1**). There is no evidence for the direct MYB-mediated regulation of camalexin biosynthesis genes downstream of IAOx. The identification of the possible role of MYB51 and MYB122 in the activation of *CYP71B15* or *CYP71A13* in complex with other, yet to be identified transcription factors, is anticipated in the future.

## Experimental Procedures

### *Arabidopsis* Lines Used in this Study

The *Arabidopsis* loss-of-function mutants used in this study are all in the Columbia-0 (Col-0) genetic background. The T-DNA insertion mutants for *MYB34*, *MYB51*, *MYB122* have been previously described and are *myb34* [*At5g60890*; WiscDsLox424F3; (Frerigmann and Gigolashvili, 2014), *myb51/hig1* (*At1g18570*; GK228B12; Gigolashvili et al., 2007), and *myb122-2* (*At1g74080*; WiscDsLoxHs206_04H; Frerigmann et al., 2014). The multiple mutants were generated and described by Frerigmann and Gigolashvili (2014).

The ethanol-inducible overexpression line *Alc::PaNie_Dc_* (Rauhut et al., 2009) and the *pMYB51::GUS* reporter line (Gigolashvili et al., 2007) were generated as described.

### Biotic and Abiotic Treatments of *Arabidopsis* Leaves

For treatment with AgNO_3_, plants were grown for six weeks under short-day conditions. Pots with five plants were sprayed with AgNO_3_ or MOCK and harvested after 18 h in the dark [AgNO_3_ (5 mM AgNO_3_ + 0.02% Silver); MOCK (0.02% Silver)].

Expression of the NEP1-like protein in *Alc::PaNie_Dc_* plants was induced by spraying with ethanol (2%) or with water for the MOCK samples. Samples were harvested at four different time points (0, 60, 150, and 300 min).

For wounding experiments, detached leaves of 6-week-old Col-0 plants were heavily crushed with forceps on the whole leaf, additionally strongly wounded with a scalpel and stored in a petri dish with wet paper tissue till harvest. After 0, 1, 5, 10, 30, 120, and 300 min leaves were frozen in liquid nitrogen and subsequent directed for RNA isolation and gene expression analysis by qRT-PCR. Wounding and storage for different time points had no effect on *ACTIN2* levels.

Five-week-old plants were infected with a 6 μL droplet of *B. cinerea* spores (2 × 10^6^ spores/μL in LB-media) or LB-media as MOCK. After infection, plants remained under short-day conditions but with a relative humidity of about 100%. Samples were harvested at different time points (0, 40, 88 h) and fixed immediately with ice-cold acetone. GUS staining was performed overnight at 37°C. Histochemical localisation of GUS in transgenic plants harboring the *pMYB51::uidA* construct was performed as described Gigolashvili et al. (2007).

### UV-Treatment, Metabolite Feeding and Camalexin Measurement

For UV induction, leaves were cut at the base of the petioles and placed on wet tissue paper under a UV-lamp (Desaga UV-VIS, = 254 nm, 8 W) at a distance of 20 cm and were irradiated for 2 h (Mucha et al., 2015). Camalexin extraction and HPLC-analysis was performed essentially as previously described (Schuhegger et al., 2006). Leaves were extracted twice in 200 μl MeOH/H_2_O (4:1; v/v) at 65°C for 30 min. Combined extracts were centrifuged at 17,000 *g* for 15 min and analyzed by reverse phase HPLC (LiChroCART 250-4, RP-18, 5 μm, Merck; 1 mL⋅min^-1^; MeOH/H_2_O (1:1; v/v) for 2 min, followed by a 10 min linear gradient to 100% MeOH, followed by 3 min 100% MeOH). Camalexin was quantified using a Shimadzu F-10AXL fluorescence detector (318 nm excitation, 370 nm emission) and by UV absorption at 318 nm applying a calibration curve with authentic standard. For intermediate feeding leaves were detached at the petiole after 2 h UV treatment and incubated in 400 μl 0.25 mM precursor solution or water for an additional 16 h.

### RNA Extraction and qRT-PCR

Total RNA extraction and qRT-PCR analysis were as described by Frerigmann and Gigolashvili (2014). The relative quantification of expression levels was performed using the comparative delta Ct method, and the calculated relative expression values were normalized to that of *ACTIN2* and compared with the expression level in untreated WT plants (Col-0 = 1). When not specified in the figure legend, three technical replicates and three biological replicates from independently grown plants were analyzed (for primer sequences see Supplementary Table S3).

### Plant Growth Conditions

Seeds of *A. thaliana* ecotype Col-0 and mutant lines were stratified for 2–7 days in the dark at 4°C to break seed dormancy. Plants were grown in growth cabinets with a light/dark cycle of 8 h/16 h and a day/night temperature of 21°C/18°C, 40% humidity and a mean photon flux density of 150 μmol m^-2^ s^-1^. A minimum of 100 mg rosette material was harvested from 6-week-old plants, immediately frozen in liquid nitrogen and kept at –80°C until RNA extraction or metabolite analysis.

### Reporter Construction for Transient Co-transformation Experiments

The promoter regions of *CYP71B15* (*At3g26830*; from –1,593 to +58 bp) and *CYP71A13* (*At1g73500*; from –2,124 to +42 bp) were amplified from genomic DNA of *Arabidopsis* plants and cloned into the *pEntry* TOPO vector (Invitrogen). The construction of the *CYP79B2* promoter was performed as described (Gigolashvili et al., 2007). The corresponding primer sequences are listed in Supplementary Table S4. The binary plant transformation vector *pGWB3i* containing an intron within the *uidA* gene was used to drive *Agrobacterium*-mediated expression of *uidA* from these promoters and *pGWB3i* was recombined with the pEntry Topo vectors containing the promoter of interest using LR reactions (Invitrogen). The final *pCYP71B15::uidA*, *pCYP71A13::uidA* and *pCYP79B2::uidA* clones in *pGWB3i*, as well as *p35S::MYB34*, *p35S::MYB51*, *p35S::MYB122*, and *p35S:WRKY33* in *pGWB2* were used to transform the supervirulent *Agrobacterium tumefaciens* strain LBA4404.pBBR1MCS.virGN54D as described by Berger et al. (2007).

## Conflict of Interest Statement

The authors declare that the research was conducted in the absence of any commercial or financial relationships that could be construed as a potential conflict of interest.

## Footnotes

1 http://atted.jp/

2 http://bar.utoronto.ca/efparabidopsis/cgi-bin/efpWeb.cgi
