# Supplementary material for: The role of MYB34, MYB51 and MYB122 in the regulation of camalexin biosynthesis in Arabidopsis thaliana
Source: Front Plant Sci. 2015 Aug 25;6:654. doi: 10.3389/fpls.2015.00654 (PMC4548095; doi:10.3389/fpls.2015.00654)
Supplement: Supplementary file 1 [file Data_Sheet_1.DOCX]

**Supplementary Material**

**Supplementary Tables**

**Table S1: Selected co-expressed genes of *MYB122*** *from* [*www.atted.jp*](http://www.atted.jp) ([Obayashi et al., 2009](#_ENREF_31))

|  | Locus[*](http://atted.jp/help/term.shtml#locus) | Alias[*](http://atted.jp/help/term.shtml#function) (Short description) | Function[*](http://atted.jp/help/term.shtml#function) | Reliability | **Ath c5.0 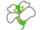 At1g74080** |
| --- | --- | --- | --- | --- | --- |
|  | [At2g30770](http://atted.jp/cgi-bin/coex_list.cgi?gene=817628) | CYP71A13 | camalexin biosynthesis; conversion of IAOx to IAN | ☆☆☆ | **42.7** |
|  | [At2g30750](http://atted.jp/cgi-bin/coex_list.cgi?gene=817626) | CYP71A12 | camalexin biosynthesis; conversion of IAOx to IAN | ☆☆☆ | **45.3** |
|  | [At4g39950](http://atted.jp/cgi-bin/coex_list.cgi?gene=830154) | CYP79B2 | camalexin/IG biosynthesis; conversion of Trp to IAOx | ☆☆ | **64.5** |
|  | [At3g26830](http://atted.jp/cgi-bin/coex_list.cgi?gene=822298) | CYP71B15  (PAD3) | camalexin biosynthesis | ☆☆☆ | **78.7** |
|  | [At3g54640](http://atted.jp/cgi-bin/coex_list.cgi?gene=824629) | TSA1 | trp biosynthesis/IG/camalexin biosynthesis | ☆☆ | **136.1** |
|  | [At1g18570](http://atted.jp/cgi-bin/coex_list.cgi?gene=838438) | MYB51 | MYB regulator of IG biosynthesis | ☆☆☆ | **285.8** |
|  | At5g05730 | ASA1 | trp biosynthesis/IG/camalexin biosynthesis | ☆☆ | **309.0** |

**Table S2: Selected co-expressed genes of *MYB51*** *from* [*www.atted.jp*](http://www.atted.jp) ([Obayashi et al., 2009](#_ENREF_31))

|  | Locus[*](http://atted.jp/help/term.shtml#locus) | Alias[*](http://atted.jp/help/term.shtml#function) (Short description) | Function[*](http://atted.jp/help/term.shtml#function) | Reliability | **Ath c5.0 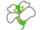 At2g30770** |
| --- | --- | --- | --- | --- | --- |
|  | At5g05730 | ASA1 | trp biosynthesis/IG/camalexin biosynthesis | ☆☆ | **141.2** |
|  | At3g26830 | CYP71B15  (PAD3) | camalexin biosynthesis | ☆☆☆ | 164.1 |
|  | At2g30750 | CYP71A12 | camalexin biosynthesis; conversion of IAOx to IAN | ☆☆☆ | **190.2** |
|  | At3g54640 | TSA1 | trp biosynthesis/IG/camalexin biosynthesis | ☆☆ | **204.5** |
|  | [At4g39950](http://atted.jp/cgi-bin/coex_list.cgi?gene=838438) | CYP79B2 | camalexin/IG biosynthesis; conversion of Trp to IAOx | ☆☆ | **259.1** |
|  | At5g17990 | TRP1 | trp biosynthesis/IG/camalexin biosynthesis | ☆☆ | **416.0** |

**Table S3.** Primer sequences for qPCR analysis

| **Oligonucleotide name** | **Gene annotation** | **Oligonucleotide sequence 5´-3´** |
| --- | --- | --- |
| Actin_RL_Fw | At3g18780 | ATGGAAGCTGCTGGAATCCAC |
| Actin_RL_Rv |  | TTGCTCATACGGTCAGCGATG |
| MYB34_RL_Fw | At5g60890 | CACGACTGTCGATAATTTTGGGTT |
| MYB34_RL_Rv |  | CATATTGTCATCTTCGTTCCAGGA |
| MYB51_RL_Fw | At1g18570 | CTACAAGTGTTTCCGTTGACTCTGAA |
| MYB51_RL_Rv |  | ACGAAATTATCGCAGTACATTAGAGGA |
| MYB122_sh_RL_RV | At1g74080 | AACTTCATTGATCGGCGTCAC |
| MYB122_sh_RL_Fw |  | ACCTCTTCGAATCTCCCCATC |
| CYP71A13_RL_Fw | At1g73500 | AATCTGAACTACATCCCATTCGGA |
| CYP71A13_RL_Rv |  | TTAGATCAGGTTGATCCCCATTTG |
| CYP71B15_RL_Fw | At3g26830 | CGTTTGGATCGTGTAGGAGAATATGT |
| CYP71B15_RL_Rv |  | TCTTCCCCATTGATGTCTTTG |
| CYP79B2_RL_Fw | At4g39950 | AACAAAAAGAAACCGTATCTGCCAC |
| CYP79B2_RL_Rv |  | TCCTAACTTCACGCATGCTATCTC |
| CYP79B3_RL_Fw | At2g22330 | CTCCTTCTTCCTTGCAAATGGA |
| CYP79B3_RL_Rv |  | GAGAATCATCAAGAAGCAAAGGG |
| PaNie_RL_Fw | Rauhut et al. 2009 | GCCGTGATCAACCATGATGCTGT |
| PaNie_RL_Rv |  | TTGTAGCCACTGTGAGCCGATGCC |

**Table S4.** Primer sequences for Promotor:*uidA* construction

| **Oligonucleotide name** | **Gene annotation** | **Oligonucleotide sequence 5´-3´** |
| --- | --- | --- |
| pPAD3_attB1_Fw | AT3G26830 | GGGACAAGTTTGTACAAAAAAGCAGGCTTC  TATAATTGTGATTCCACTCTTGTGTATTGT |
| pPAD3_attB2_Rv |  | GGGACCACTTTGTACAAGAAAGCTGGGTC  GAGGACGAGGAAACAGAGGAAAAC |
| pCYP71A13_attB1_Fw | AT1G73500 | GGGACAAGTTTGTACAAAAAAGCAGGCTTC  GGGAATCAGTGAGGACAAGATAATTG |
| pCYP71A13_attB2_Rv |  | GGGACCACTTTGTACAAGAAAGCTGGGTC  GGTTATGAGGGTCGTTAAGCACAA |

**Supplementary Figures**


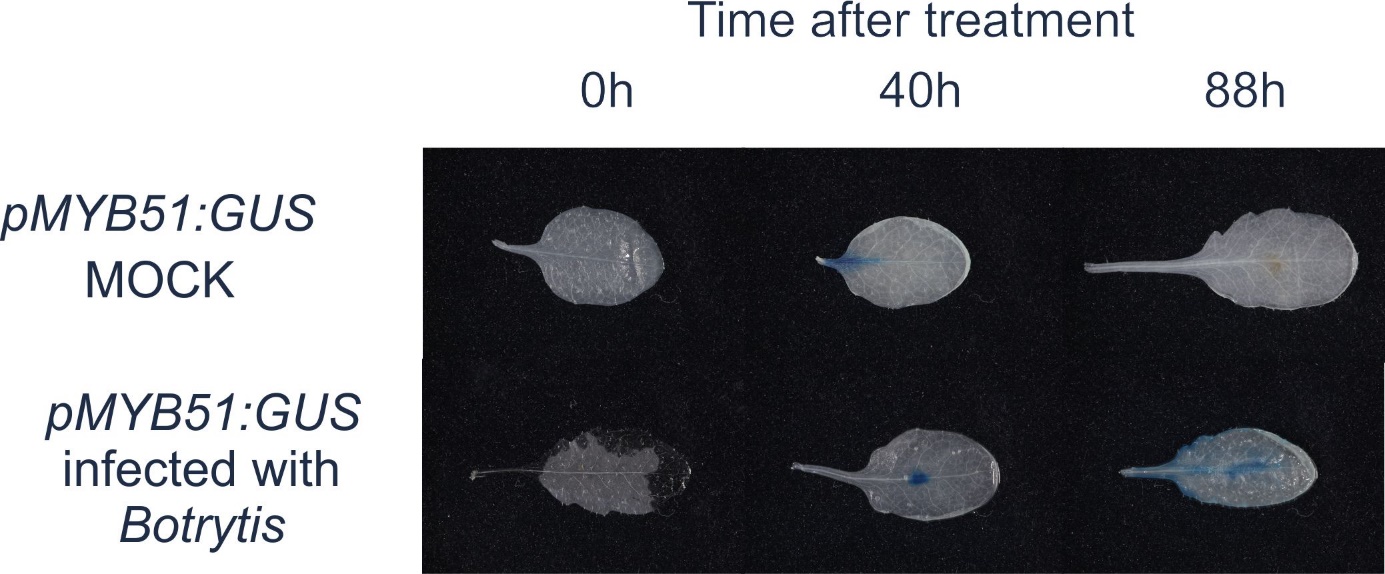


Fig. S1. Local induction of *MYB51* by *Botrytis cinerea* infection

Leaves of 5-week-old *pMYB51::GUS* plants were inoculated with 6 µL spore solution (1 × 10^6^) in LB or with only LB as a MOCK treatment. Leaves were harvested at four different time points followed by GUS staining to visualise the activity of the *MYB51* promoter. Black circles indicate the position on the leaf where the spore solution was applied.


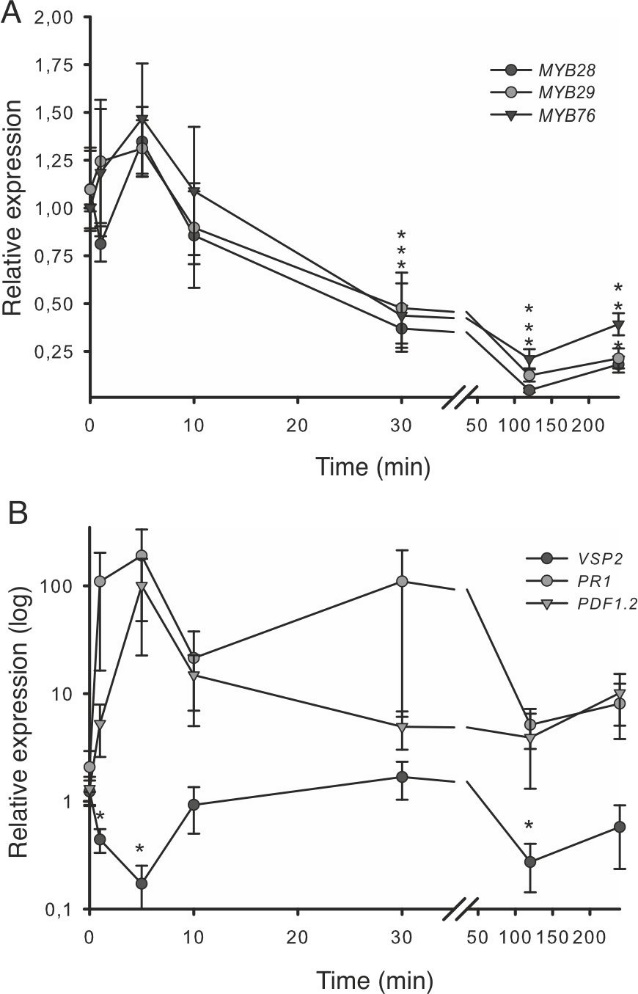


**Fig. S2. Wounding response of *HAG/MYB* and hormone marker genes in leaves**

Detached leaves of 6-week-old Col-0 plants grown under short day conditions were strongly wounded with a scalpel. Leaves were harvested after 1 min, 5 min, 10 min, 30 min, 120 min and 300 min and processed for transcript analysis by qPCR. Relative transcript levels for (**A**) *HAG/MYB* (*MYB28*, *MYB29,* *MYB76*) and (**B**) hormone marker genes for jasmonate (*VSP2*), salicylic acid (*PR1*) and ethylene/jasmonate (*PDF1.2*) are shown for wounded vs. unwounded leaves (time-point 0 = 1 min). Data are means ± SE from three independent cultivations each with two biological replicates (n = 6). Values marked with asterisks are significantly different from the 0 time point (Student’s *t*-test; *p* < 0.05).

**
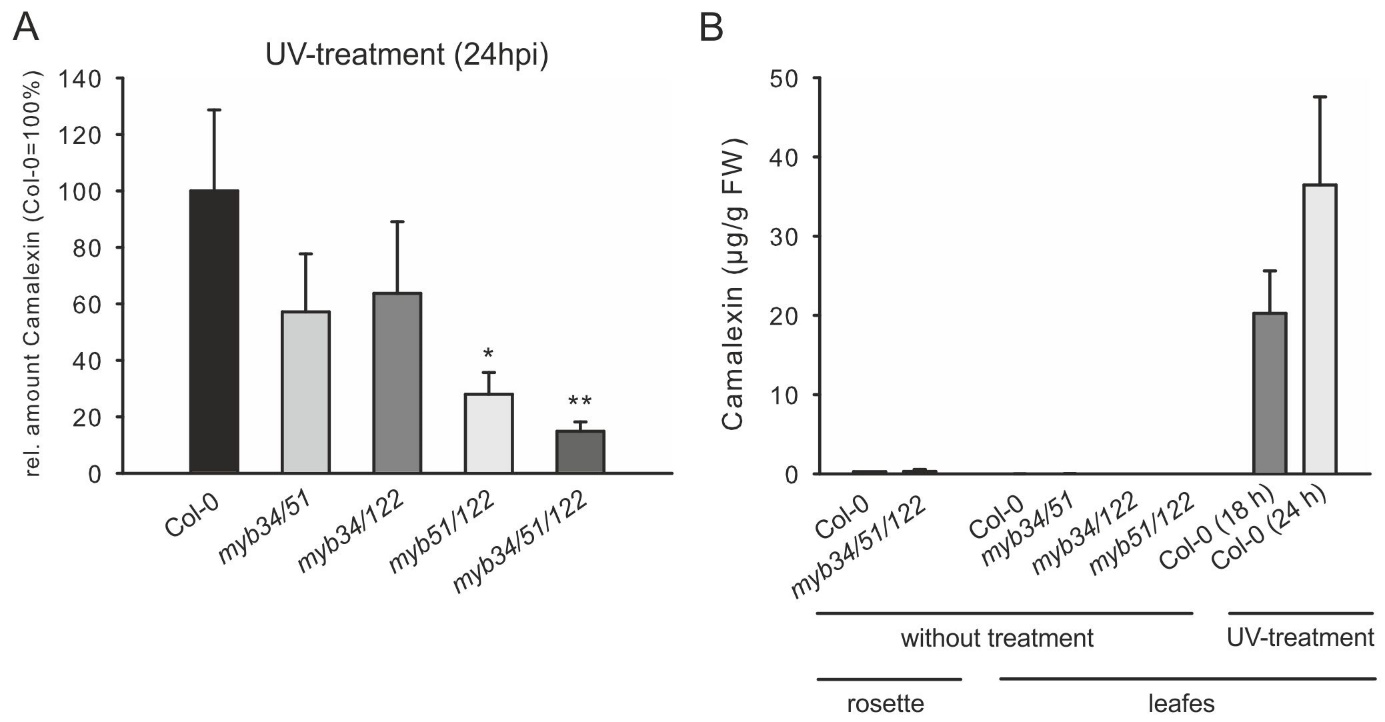
**

**Fig. S3. The UV-dependent induction of camalexin is impaired in *myb* mutants**

The relative amount of camalexin in Col-0 and the double and triple *myb* mutants 24 h after UV treatment is shown (Col-0 = 100%) (**A**). Values marked with asterisks are significantly different from those of control plants (Student’s *t*-test; * *p* < 0.05; ** *p* < 0.01). **Fig. B** presents absolute levels (µg/g FW) of camalexin in Col-0 and the *myb* mutants without treatment or in Col-0 18h and 24h after UV treatment. Data are means ± SE from two independent cultivations with six biological replicates (n = 12; without treatment: n = 3).
